# Risk Assessment of Potentially Toxic Heavy Metals in Wheat (*Triticum aestivum* L.) Grown in Soils Irrigated with Paper Mill Effluent

**DOI:** 10.3390/toxics13060497

**Published:** 2025-06-13

**Authors:** Mohssen Elbagory, Amal Zayed, Nagwa El-Khateeb, Sahar El-Nahrawy, Alaa El-Dein Omara, Ibrahim Mohamed, Marwa Yasien Helmy Elbyaly, Mahmoud El-Sharkawy, Jogendra Singh, Ana Dzaja, Boro Mioč, Ivan Širić

**Affiliations:** 1 Health Specialties, Basic Sciences and Applications Unit, Applied College, King Khalid University, Mohayil Asir Abha 61421, Saudi Arabia; 2 Educational Psychology Department, Faculty of Education, Kafrelsheikh University, Kafr El-Sheikh 33516, Egypt; 3 Agricultural Botany Department, (Agricultural Microbiology), Faculty of Agriculture, Kafrelsheikh University, Kafr El-Sheikh 33516, Egypt; 4 Soil Microbiology Research Department, Soils, Water, and Environment Research Institute (SWERI), Agriculture Research Center (ARC), Giza 12112, Egyptalaa.omara@yahoo.com (A.E.-D.O.); 5 Department of Soil and Water Sciences, Faculty of Agriculture, Benha University, Moshtohor, Toukh 13736, Egypt; 6 Department of Home Economics, Faculty of Specific Education, Kafrelsheikh University, Kafrelsheikh 33516, Egypt; 7 Soil and Water Department, Faculty of Agriculture, Tanta University, Tanta 31527, Egypt; 8 Research and Development Division, Agriculture and Environmental Science Academy, Haridwar 249404, India; 9 Institucija SAN Eko d.o.o., Buzinski prilaz 10, 10010 Zagreb, Croatia; 10 University of Zagreb Faculty of Agriculture, Svetosimunska 25, 10000 Zagreb, Croatia; bmioc@agr.hr

**Keywords:** health risk assessment, heavy metals, modeling analysis, paper mill effluent

## Abstract

Unregulated irrigation with partially industrial effluents may lead to heavy metal contamination in crops and pose significant human health risks, especially in developing countries like India. Therefore, the present study aimed to quantify six heavy metals (Cd, Cr, Cu, Fe, Mn, and Zn) in soil and wheat irrigated with paper mill effluent, assess plant responses, and evaluate associated health risks for consumers. For this, a field study was conducted across ten sites (five effluent-irrigated, five borewell-irrigated as control), analyzing soil and wheat tissues for metal concentrations and calculating risk indices including bioaccumulation factor (Bf), translocation factor (Tf), Dietary Intake of Metals (DIM < 1), Health Risk Index (HRI < 1), and Target Hazard Quotient (THQ < 1). Results indicated high concentrations of Cd and Cr in effluent-irrigated soils and wheat tissues (root > stem > leaves > grains) compared to control sites, with some values exceeding permissible limits. Although the THQ values for heavy metals were below 1, indicating a low immediate health risk, concentrations of Cd and Cr in both soil and crop tissues exceeded acceptable safety standards. This study provides empirical evidence supporting the need for effluent treatment and policy interventions to mitigate agricultural contamination from the use of industrial effluents and protect public health.

## 1. Introduction

Heavy metal pollutants are persistent in the environment and tend to accumulate in soil and water systems, subsequently entering the food chain and posing serious health risks to humans [1]. These pollutants are primarily introduced through industrial processes and the discharge of untreated or inadequately treated sewage, which affect both surface and groundwater bodies [2]. The chemical composition of water is a fundamental determinant of ecosystem health and water safety, directly influencing aquatic biodiversity and human survival [3]. Irrigation with wastewater, particularly from industrial and domestic sources, alters soil physicochemical properties and promotes the accumulation of toxic elements. This accumulation can result in reduced crop productivity and elevated health risks [4,5]. Heavy metals such as Lead (Pb), Mercury (Hg), Cadmium (Cd), Arsenic (As), Chromium (Cr), Nickel (Ni), Copper (Cu), Zinc (Zn), Manganese (Mn), Cobalt (Co), and Silver (Ag) are frequently detected in industrial effluents, including wastewater discharged from paper mills. At elevated concentrations, these elements pose significant ecological and health hazards, affecting crops, soil quality, aquatic life, and human well-being [6,7].

Heavy metals pose significant health risks to humans due to their toxicity and persistence in the environment [8]. These metals can accumulate in vital organs, causing disorders including kidney damage, neurological deficits, and carcinogenic effects [9]. Paper mill effluents often contain elevated levels of these heavy metals, originating from bleaching agents and additives used in the pulping process [10]. When released untreated into agricultural fields via wastewater irrigation, these contaminants can bioaccumulate in soils and crops, posing a direct threat to food safety and public health [11]. Therefore, understanding this context is essential for assessing the environmental and health risks associated with wastewater reuse.

Wheat (*Triticum aestivum* L.) is a major staple cereal crop globally, especially in developing countries, where it contributes approximately 20% of the per capita calorie and protein intake. Beyond its nutritional value, wheat plays a critical role in promoting human health [12]. However, agricultural sustainability is increasingly threatened by environmental degradation, particularly due to the contamination of soil and water resources with heavy metals arising from both natural and anthropogenic activities. The toxicological impacts of prolonged exposure to heavy metals such as Pb, Co, Ag, Ni, Cu, and Cr include liver and kidney dysfunction, neurotoxicity, and other chronic disorders [13], highlighting the critical need for risk assessment in agroecosystems subjected to industrial pollution. The paper manufacturing industry generates substantial volumes of solid and liquid waste, including sludge, bark residues, and colored effluents. If inadequately treated, these by-products contribute significantly to environmental contamination. Paper mill wastewater often contains a complex mixture of heavy metals due to the chemicals used during production, which can pollute both aquatic and terrestrial ecosystems [3]. In particular, the Star Paper Mill located in the Saharanpur District of Uttar Pradesh has a production capacity of approximately 2500 tons per day [14]. Waste from this facility is discharged into the Kali River through an open canal and is frequently used by local farmers for crop irrigation.

This study addresses a critical regional concern where untreated paper mill effluents are routinely used for irrigation, particularly in the Saharanpur district of Uttar Pradesh, India. While the health risks are well recognized, empirical data on heavy metal accumulation in wheat—a dietary staple vital to nutritional security in this region—remain limited. Furthermore, although treated effluent is widely used in agriculture, no studies have systematically assessed the risks of potentially toxic heavy metal accumulation in soil and wheat crops irrigated with paper mill wastewater in this region. A recent study by Qu et al. [15] also highlighted the importance of contextual risk assessments under effluent irrigation practices. Therefore, this study aims to fill this research gap by evaluating contamination levels and assessing associated health risks. Therefore, the objectives of this study are to: (1) determine the concentration and spatial distribution of selected heavy metals in soil and wheat plant tissues; (2) analyze pollution indices such as the bioaccumulation factor, and translocation factor, alongside evaluate the effects of these metals on plant growth parameters; (3) assess potential human health risks linked to exposure to these metals using established risk assessment methodologies.

## 2. Materials and Methods

### 2.1. Study Area and Sample Collection

This study was conducted in the suburban region of Saharanpur District, Uttar Pradesh, India, where agriculture forms the primary livelihood of the local population (29°55′34.6″ N and 77°35′31.4″ E). The area lies adjacent to Star Paper Mill Ltd., a major industrial unit. The mill discharges large volumes of untreated or partially treated wastewater into 3 km-long and 3 m-wide drainage channels, which ultimately connect to the Kali River. Due to limited water resources, local farmers commonly use this effluent for crop irrigation. To assess the impact of this practice, two groups of agricultural fields were selected: five irrigated with borewell water (control) and five with paper mill effluent (exposed), totalling ten sites. Sampling was carried out during two wheat-harvesting seasons in April 2023 and April 2024. From each field, three sub-samples of soil and plant material were collected and pooled, yielding one composite sample per field. Thus, for each season, ten pooled samples per matrix (soil and plant) were obtained, totalling 20 samples per group over both seasons. Soil samples were collected from the top 0 to 20 cm layer using a stainless-steel auger at three random points within each field and then pooled to form one composite sample. Similarly, wheat plants at maturity were uprooted from the same locations, and roots, shoots, and grains were separated for analysis. Soil and plant tissue samples intended for heavy metal analysis were dried, ground, and stored in labeled polyethylene bags until analysis. Fresh leaf samples were used immediately for pigment (chlorophyll) analysis to avoid degradation.

### 2.2. Soil, Water, Effluent Collection and Analysis Processing

For this study, selected physicochemical and heavy metal parameters were analyzed in soil, irrigation water, and plant samples. Composite soil samples (~2 kg) were collected from the root zone (0–15 cm) using a stainless-steel auger, placed in polyethylene zip-lock bags, and transported to the laboratory. Soil samples were air-dried, ground, and sieved (2 mm mesh) before analysis. Soil pH and EC were measured in a 1:2.5 soil-to-water suspension using a microprocessor-based multiparameter meter (Model 1611, ESICO Int., Parwanoo, Himachal Pradesh, India). Organic matter (OM) content was determined by the Walkley and Black titration method, involving oxidation with potassium dichromate and sulfuric acid, followed by back-titration with ferrous ammonium sulfate. Total Kjeldahl Nitrogen (TKN) was analyzed using the classical Kjeldahl digestion–distillation method, involving sulfuric acid digestion, followed by neutralization and steam distillation. Available Phosphorus (P) was extracted using Olsen’s method and quantified by UV–Vis spectrophotometry (Cary 60, Agilent Technologies, Santa Clara, CA, USA) at 882 nm. Exchangeable Potassium (K) was extracted using ammonium acetate and determined using a flame photometer (Model 1382, ESICO, Parwanoo, Himachal Pradesh, India). Sodium (Na) was also measured using the same flame photometric method. Heavy metals (Cd, Cr, Cu, Fe, Mn, Zn) were extracted using a tri-acid digestion method (HNO_3_:H_2_SO_4_:HClO_4_ = 5:1:1 *v*/*v*) and quantified by atomic absorption spectroscopy (AAS; Analyst 800, PerkinElmer, Waltham, MA, USA), using metal-specific hollow cathode lamps and appropriate wavelengths.

Similarly, water samples (borewell and treated paper mill effluent) were collected in pre-cleaned polyethylene bottles, acidified with HNO_3_ (pH < 2), and stored at 4 °C until analysis. pH, EC, and TDS were measured immediately using the same multiparameter meter as for soil. Biological Oxygen Demand (BOD) was estimated using a 5-day incubation method followed by titration using Winkler’s method with a digital DO meter (Model 1801, ESICO, Parwanoo, Himachal Pradesh, India). Chemical Oxygen Demand (COD) was determined by the standard open reflux dichromate method using sulfuric acid and potassium dichromate. TKN was determined following the same digestion–distillation protocol used for soil. P, Na, and K were also quantified as described above. Heavy metals (Cd, Cr, Cu, Fe, Mn, Zn) in water samples were analyzed following acid digestion with concentrated HNO_3_ and H_2_SO_4_ (3:1 ratio) and measured using AAS. All water and soil analytical procedures adhered to the standard protocols of APHA [16] and AOAC (2016) [17].

For plant analysis, fresh leaf samples were collected at the vegetative stage for physiological assessment, while root, stem, leaf, and grain samples were harvested at maturity. Chlorophyll content was determined by extracting fresh leaves in 80% acetone and measuring absorbance at 645 nm and 663 nm using a spectrophotometer. Chlorophyll a, b, and total content were calculated using Arnon’s equations [18]. Morphological parameters including plant height, leaf length, leaf width, and leaf area were recorded using standard agronomic protocols. Heavy metal concentrations in plant tissues were measured after oven drying at 70 °C, grinding, and digestion with HNO_3_:HClO_4_ (4:1 *v*/*v*). The digested samples were analyzed by AAS under the same conditions used for soil and water. The heavy metal analysis was carried out following the standard methodologies and protocols as adopted by Shah et al. [19].

### 2.3. Plant Growth Indices and Accumulation Factors

The Leaf Area Index (LAI) was determined to evaluate plant growth and canopy development using leaf length and width measurements. This index is an important indicator of photosynthetic capacity and biomass productivity [20,21].

(1) $$LA\left({cm}^{2}\right)=k\times (LL\times LW)$$

where *k* is the correction factor (0.75), *LL* represents leaf length (cm), and *LW* denotes leaf width (cm), respectively.

To evaluate the extent of growth, heavy metal uptake, and movement within the wheat plant system, several indices were employed, including the Bioaccumulation Factor (Bf), Translocation Factor (Tf), and Leaf Area Index (LAI). These indices help quantify metal accumulation from soil to plant tissues and assess physiological responses related to growth under different irrigation conditions. The Bioaccumulation Factor (Bf) was used to assess the capacity of wheat to accumulate heavy metals from soil into its tissues, indicating the extent of metal uptake relative to soil concentration [22]. The Translocation Factor (Tf) indicates the efficiency of heavy metal movement from roots to aerial plant parts. A Tf value ≤ 1 signifies limited translocation, while Tf > 1 reflects effective metal transport to shoots and grains [23].

(2) $$Bioaccumulation\;factor\;(Bf)=\frac{HM\;in\;wheat\;tissues}{HM\;in\;the\;soil\;sample}$$

(3) $$Translocation\;factor\;(Tf)=\frac{HM\;in\;the\;aerial\;tissues}{HM\;in\;root\;tissues}$$

where HM refers to heavy metal concentration in soil or plant tissue (mg/kg dwt.).

### 2.4. Health Risk Assessment

To evaluate potential health risks, dietary intake of metal (DIM), hazard risk index (HRI), and target hazard quotient (THQ) were computed for wheat grain, using established risk assessment models. In this, Equations (4) and (5) were used to compute HRI and DIM values as given below:

(4) $$HRI=\frac{DIM}{RfD}$$

(5) $$DIM=SL\times \frac{HMc}{Bw}$$

where RfD, SL, HMc, and Bw represent oral reference dose, the serving of wheat (dried weight), heavy metal concentration, and body weight of consumers (70 kg for adults and 52 kg for children), respectively. Additionally, THQ was used to evaluate the health risk of heavy metal accumulation in wheat as per the following model (Equation (6)):

(6) $$THQ=10^{-3}\times \frac{Ef\times Ed\times Ir\times HM}{Bw\times Cp\times RfD}$$

where 10^−3^ is the conversion factor, Ef is the exposure frequency, Ed is the exposure duration (365 days), Ir represents the wheat ingestion rate (0.30 kg/day), HM is heavy metal concentration in the wheat sample (mg/kg), Bw corresponds to the average body weight, Cp is the consumption period (21,900 days for adults and 5475 days for children), and RfD is the reference dose in suggested by USEPA terms of mg/kg/day (Cd: 1.0 × 10^−3^; Cu: 4.0 × 10^−2^; Cr: 5.0 × 10^−3^; Fe: 7.0 × 10^−1^; Mn: 1.4 × 10^−2^, and Zn: 3.0 × 10^−1^). Further, the combined toxicity of heavy metal intake from wheat was calculated as per Equation (7):

(7) ∑THQ=THQ(Cd+Cu+Cr+Fe+Mn+Zn)

### 2.5. Statistical Analysis

Data analysis was performed using Microsoft Excel 2019 (Microsoft Corp., Redmond, Washington, USA). A one-way analysis of variance (ANOVA) was applied to assess significant differences among water sources, irrigated soils, and wheat plant growth and yield parameters. To assess differences between irrigation treatments, Tukey’s Honestly Significant Difference (HSD) post hoc test was applied to evaluate comparisons among plant tissue types for heavy metal concentrations. Statistical significance was determined at a probability level of *p* < 0.05.

## 3. Results and Discussion

### 3.1. Characteristics of Water, Effluent, and Agricultural Soil

The physicochemical characteristics of borewell water and paper mill effluent, along with their effects on irrigated soils, are presented in Table 1 and Table 2. Statistically significant differences (*p* < 0.05) were observed between borewell water and paper mill effluent across all measured parameters, as determined by a two-tailed Student’s *t*-test. These results indicate that the effluent contains higher concentrations of key pollutants, which may influence soil quality and pose risks to agricultural sustainability. It was evident that both water sources had acceptable pH values, but the effluent was more alkaline than borewell water. The effluent showed higher electrical conductivity and total dissolved solids, indicating increased salinity [24]. It also contained excessive BOD and COD, exceeding permissible limits and implying a high organic load [25,26]. Nutrient concentrations such as TKN and P were significantly high in the effluent, suggesting potential fertility benefits but posing risks of soil pollution [27]. The effluent also exhibited high Na and K levels, suggesting the potential for sodicity-induced soil degradation [28,29]. Among heavy metals, Cr slightly exceeded disposal limits, while Fe and Mn also surpassed thresholds; although Cd, Cu, and Zn were within permissible levels, their concentrations were substantially higher in effluent than in borewell water [30,31].

Statistically significant differences (*p* < 0.05) were observed in all measured properties between soils irrigated with effluent and those irrigated with borewell water, as determined by a two-tailed Student’s *t*-test (Table 2). Soil analyses showed higher pH and EC under effluent irrigation, indicating increased alkalinity and salinity. OM content nearly doubled in effluent-irrigated soil, attributed to the organic load [32]. Other parameters such as TKN, P, Na, and K were significantly higher in these soils, suggesting nutrient enrichment that may enhance fertility but risks imbalance or toxicity over time [33]. Effluent-irrigated soils showed markedly elevated levels of Cd, Cr, Cu, Fe, Mn, and Zn, indicating accumulation risks and potential long-term contamination [34,35]. While effluent use may boost fertility, its untreated application poses serious environmental pollution and agronomic risk.

Several recent studies have reported comparable findings regarding soil and wastewater characteristics. Out of them, Khan et al. [4] observed pH, EC, organic matter, and available P and K within typical ranges. Nowwar et al. [13] analyzed wastewater and recorded key parameters such as pH, EC, TDS, COD, BOD, NH_3_-N, and concentrations of heavy metals (Cd, Cr, Cu, Fe, Mn, Zn). Also, Nepal [36] evaluated heavy metal accumulation in mustard under different cultivation practices, highlighting the presence of Cd, Zn, Cu, Mo, Pb, and Ni. These studies support the current findings and reflect the variability in soil and crop contamination patterns. To mitigate heavy metal contamination in effluent-irrigated systems, widely adopted remediation strategies such as phytoremediation, biochar amendment, and microbial-assisted remediation should be explored. These techniques offer practical and sustainable options for reducing metal bioavailability and uptake. Recent advancements, as discussed in Wang et al. [37] highlight the effectiveness of integrated approaches combining biological and physicochemical methods.

### 3.2. Effects on Wheat Plant Attributes

The results presented in Table 3 indicate the effects of paper mill effluent irrigation on wheat growth and physiological parameters compared to borewell water (control). In this, all measured variables, i.e., total chlorophyll content, plant height, leaf length, leaf width, and leaf area showed statistically significant increases under effluent treatment (*p* < 0.05). Specifically, chlorophyll content significantly increased in effluent-treated plants compared to the control, indicating enhanced photosynthetic potential. This increase could be attributed to the presence of essential nutrients such as N and Mg in effluent, which are known to support chlorophyll biosynthesis [38]. An increase in chlorophyll concentration often correlates with improved photosynthetic efficiency and biomass accumulation. Plant height also improved under effluent irrigation relative to borewell-irrigated plants (86.28 ± 4.17 cm), suggesting better vegetative growth. Previous studies have linked such growth stimulation to the organic load and nutrient availability in treated wastewater [39]. Similarly, leaf morphological traits improved: leaf length increased from 17.52 ± 0.59 cm to 22.08 ± 0.78 cm, and leaf width rose from 0.32 ± 0.03 cm to 0.38 ± 0.05 cm in effluent-irrigated plants. The combined effect of these increases led to a marked expansion in leaf area (6.37 ± 1.05 cm^3^ vs. 4.17 ± 0.50 cm^3^), which supports higher light capture and transpiration surface, thus promoting plant productivity [40]. While these short-term benefits are useful, long-term application of paper mill effluent may lead to salt accumulation or heavy metal toxicity, potentially impacting soil health and plant performance [41,42]. Previously, Malik [43] evaluated wheat plant traits under various municipal solid waste treatments and observed significant variations in leaf length, width, area, and shoot length. Similarly, Al-Huqail et al. [14] investigated the impact of heavy metal-contaminated wastewater on two rice varieties. They reported differences in plant height, chlorophyll content, and carotenoid levels. These findings align with the current study, indicating that wastewater and solid waste treatments influence plant morphological and physiological responses. Hence, controlled use and periodic monitoring are essential for sustainable irrigation practices.

### 3.3. Heavy Metal Accumulation in Wheat Tissues Under Different Irrigation Regimes

The concentrations of selected heavy metals (Cd, Cr, Cu, Fe, Mn, and Zn) in wheat tissues—including roots, stems, leaves, and grains—under two irrigation regimes (borewell water as control and paper mill effluent) are presented in Table 4. Statistical analysis using Tukey’s HSD test (*p* < 0.05) showed significant differences both among plant parts and between irrigation treatments. In this, Cd concentration was highest in roots under both treatments, with a sharp increase from 0.09 ± 0.01 mg/kg (control) to 0.21 ± 0.02 mg/kg (effluent). Although grain Cd levels remained low, the increase under effluent irrigation suggests systemic uptake and translocation [44]. In contrast, Cr levels decreased under effluent irrigation, particularly in roots and grains, which may reflect lower bioavailable Cr species in the effluent [45]. The most increases were observed for Cu, especially in roots, where levels increased from 1.72 ± 0.02 to 8.52 ± 0.02 mg/kg. Similar trends were observed in stems, leaves, and grains, indicating a strong effluent-driven accumulation gradient [46]. This suggests that certain metals are highly mobile and bioavailable in effluent-irrigated systems, affecting both edible and non-edible plant parts. On the other hand, Fe accumulation increased significantly in all plant tissues under paper mill effluent irrigation, with root concentrations tripling from 15.72 ± 0.12 to 45.76 ± 0.29 mg/kg. The grain Fe content also increased substantially, which may have implications for dietary intake and fortification potential [47]. Mn concentrations followed a similar trend where root Mn increased from 12.33 ± 0.10 mg/kg (control) to 35.34 ± 0.07 mg/kg (effluent), with statistically significant differences across all tissues, confirming enhanced Mn bioavailability in the presence of effluent [48]. Also, Zn showed substantial uptake under effluent conditions, with root concentrations rising from 15.64 ± 0.04 to 23.24 ± 0.07 mg/kg, and grain Zn doubling from 2.43 ± 0.02 to 4.17 ± 0.02 mg/kg. These increases in essential micronutrients might initially appear beneficial, but excessive accumulation poses risks of phytotoxicity and human exposure through consumption. While effluent irrigation enhanced the uptake of Cu, Fe, Mn, and Zn, the concurrent increase in Cd raises concerns over food safety. Long-term field trials and risk assessments are essential to determine the sustainability of using paper mill effluent in crop production [49].

Plants absorb heavy metals primarily through root systems via ion channels and transporters that normally mediate essential nutrient uptake [50]. Metals can enter through these pathways due to their chemical similarity to nutrient ions. Once inside roots, heavy metals translocate to shoots and leaves via the xylem [51]. Intracellularly, metals may bind to phytochelatins or metallothioneins, which sequester and detoxify them, mitigating cellular damage [52]. Thus, understanding these mechanisms clarifies how heavy metals accumulate in edible plant parts, influencing food safety and crop quality. Repeated irrigation with heavy metal-contaminated wastewater can lead to the accumulation of toxic elements in soil, altering its physicochemical properties and microbial communities [53]. Over time, this accumulation reduces soil fertility and disrupts nutrient cycling, potentially causing phytotoxicity and reduced crop yields [54]. Persistent contamination challenges soil remediation and may restrict land use. Therefore, long-term monitoring and management strategies are essential to maintain agricultural sustainability and protect public health. In a previous study, Tong et al. [55] examined heavy metal bioaccumulation and transfer in highland barley and wheat, noting that metal concentrations varied among plant parts. Cd, Cr, Ni, and Pb followed the pattern root > leaf > stem > grain, while Cu, Mn, and Zn showed different distribution trends. Grain metal concentrations varied by crop type, with wheat and barley showing distinct accumulation ranges. Al-Huqail et al. [14] similarly assessed metal accumulation in the straw and grains of two rice varieties irrigated with different water sources. They reported variable concentrations of Cd, Cr, Cu, Fe, Mn, and Zn across the samples. These studies collectively support the influence of water quality and crop type on heavy metal uptake and distribution in edible and non-edible plant parts.

### 3.4. Bioaccumulation Factor (Bf) of Heavy Metals

The bioaccumulation factor analysis showed a significantly different (*p* < 0.05) uptake and internal distribution of heavy metals in wheat tissues under borewell and paper mill effluent irrigation (Table 5). In this, Cd exhibited minimal bioaccumulation under both regimes, with values below 1 across all plant parts. In borewell-irrigated plants, Cd followed the trend: root > stem > leaves > grain, while under effluent irrigation, concentrations declined further: root > stem > leaves > grain, suggesting limited uptake and translocation due to its low mobility [56]. Also, Cr followed a similar trend, with slightly higher values in borewell-irrigated plants: root > stem > leaves > grain, while effluent irrigation led to significantly reduced Bf values: root = stem > leaves > grain, indicating low environmental bioavailability or exclusion mechanisms [57]. However, Cu showed the highest Bf in borewell-irrigated plants: root > stem > leaves > grain, indicating strong uptake and retention in roots with limited translocation, suggesting phytostabilization potential. Under effluent irrigation, Cu Bf values decreased substantially: root > stem > leaves > grain, suggesting moderate uptake with minimal grain accumulation [58]. Fe accumulation was moderate in both irrigation regimes. In this, borewell irrigation resulted in Bf values slightly above 1: root > stem > leaves > grain, indicating essential nutrient uptake with decreasing concentration along the plant height. Effluent irrigation resulted in slightly lower Fe Bf: root > stem > leaves > grain, suggesting a similar physiological distribution with minimal risk to grain quality [59].

However, Mn showed high Bf values across all plant parts in both conditions, more distinct under borewell irrigation: root > stem > leaves > grain, and slightly reduced under effluent irrigation: root > stem > leaves > grain, relating to active uptake and efficient transport. Despite its essential role, high Mn in grains may pose toxicological risks if environmental concentrations are excessive [60]. On the other hand, Zn exhibited strong accumulation under borewell irrigation: root > stem > leaves > grain, and moderately lower values under effluent: root > stem > leaves > grain, reflecting essential nutrient behavior with considerable translocation to edible parts, warranting monitoring in contaminated soils [59]. Al-Huqail et al. [14] reported that heavy metal accumulation in rice followed the order Fe > Mn > Zn > Cu > Cr > Cd, with Bf exceeding 1 in straw and remaining below 1 in grain, indicating greater accumulation in non-edible parts. Similarly, Nepal et al. [36] observed that mustard exhibited BAF values above one for Cd and Mo across all varieties, suggesting effective uptake of these metals. In contrast, Cu, Pb, Ni, and Zn showed Bf below 1, indicating limited accumulation.

### 3.5. Translocation Factor (Tf) of Heavy Metals

The Tf quantifies the movement of heavy metals from roots to aerial parts of a plant, providing information on their internal mobility and distribution. The root serves as the baseline reference for each metal’s uptake [61]. As shown in Table 6, Cd and Cr exhibited the highest mobility among the analyzed metals, with translocation patterns showing a gradual decrease in concentration from roots to stems, leaves, and grains. The upward movement, particularly into edible tissues, raises concerns regarding potential dietary exposure and associated health risks [62,63]. Similarly, Fe demonstrates moderate mobility, indicating a substantial level of absorbed Fe reaches the grain. Although it is less mobile than Cd and Cr, its movement remains significant in nutritional and physiological terms [64]. Mn displayed a comparable translocation trend but with slightly reduced values suggesting moderate mobility with partial restriction at later translocation stages [65]. Zn also exhibits moderate translocation efficiency, with decreasing Tf values from root to stem. This trend implies reasonable movement through the vascular system but limited allocation to the grain, indicating a controlled distribution mechanism [66].

In contrast, Cu shows the lowest mobility across all plant parts, with a steep decline in Tf values from root to stem, leaf, and grain. This indicates strong retention in the roots and minimal transport to aerial tissues, especially the edible grain, limiting potential dietary intake but also restricting its use in phytoremediation strategies. The observed increase in Cd and Cr, suggests specific uptake and transport mechanisms in wheat, likely influenced by soil pH and organic matter under effluent irrigation. Also, the variation in bioaccumulation and translocation factors across tissues indicates differential metal mobility and partitioning, which may be governed by metal-specific transport proteins and wheat responses. Overall, Cd, Cr, and Fe demonstrated the highest translocation efficiencies and reached the grain, whereas Cu remains largely confined to the roots. These results are useful for evaluating food safety risks and for optimizing the use of plants in soil remediation or nutrient biofortification programs [67]. Tong et al. [55] reported that both highland barley and wheat showed high Tf for Zn, Cu, and Mn from stem to grain, indicating efficient metal movement to edible parts. Wheat exhibited slightly higher Tf values than barley. Similarly, AO et al. [68] assessed soil-to-plant transfer coefficients for multiple heavy metals in crops grown near a mining site. Notable variations were observed among Zea mays, Abelmoschus esculentus, and *Arachis hypogaea*, with metals like Cu and Zn showing higher transfer rates.

### 3.6. Health Risk Assessment of Heavy Metals

The health risk assessment of heavy metal accumulation in wheat irrigated with borewell and effluent water was quantified using DIM, HRI, and THQ for both adults and children exposure groups (Table 7). For adults consuming wheat grain irrigated with borewell water, DIM values were low across all metals, with the highest for Fe and the lowest for Cd. Corresponding HRI values remained well below the risk threshold (HRI < 1), with Mn and iron showing the highest indices [60]. THQ values for all metals were negligible, with a combined ∑THQ of 0.00046, indicating no significant non-carcinogenic risks [69]. In contrast, wheat irrigated with paper mill effluent presented slightly elevated DIM values, particularly for Cu, Fe, and Mn. The HRI for Mn was the highest among the metals, approaching but not exceeding the risk threshold. Nonetheless, the overall ∑THQ for adults remained low (0.00075), suggesting that the long-term health risk from consuming these crops is minimal under current exposure levels [70]. These trends confirm that borewell irrigation poses minimal risk, while effluent irrigation requires continued monitoring due to elevated Mn and Cu levels.

Children exposure group exhibited higher DIM, HRI, and THQ values compared to adults, owing to their lower body mass and higher dietary intake relative to weight [71]. For children exposed to borewell-irrigated crops, DIM values for Fe and Mn were highest, while Cd and Cu remained low. HRI values, although elevated compared to adults, remained under the critical threshold, with Mn (0.149) and Fe being the most prominent contributors to potential health risks [72]. THQ values for children reached a combined ∑THQ of 0.00060, indicating negligible non-carcinogenic health effects [73]. However, children consuming crops irrigated with paper mill effluent had a ∑THQ of 0.00100, nearly double the borewell value. The DIM for Fe, Mn, and Cu was remarkably higher, with HRI values for Mn and Cu contributing substantially to overall risk [74]. Although these values remain below the risk threshold, they highlight the vulnerability of children to long-term exposure. Therefore, while current contamination levels do not pose immediate health threats, Mn and Cu require regular monitoring in effluent-irrigated soils to ensure long-term food safety, particularly for sensitive groups like children [75]. Although the THQ values for all metals remained below 1, Mn and Cu exhibited values approaching the threshold, particularly in children. This proximity suggests a potential risk under prolonged exposure or in scenarios of dietary variation and bioavailability changes. Therefore, even sub-threshold THQ values warrant regular monitoring and preventive interventions, especially for vulnerable groups. Policy frameworks should enforce stringent limits on heavy metal concentrations in wastewater used for irrigation, supported by regular monitoring protocols. Agronomically, practices such as selecting crop varieties with lower metal uptake, applying soil amendments (e.g., biochar and lime), and adopting phytoremediation can reduce heavy metal bioavailability [76]. Crop rotation and maintaining soil organic matter also help mitigate risks [77,78]. Farmer education on wastewater risks and safe irrigation techniques is crucial. Implementing integrated approaches combining regulation and best management practices will optimize wastewater reuse while safeguarding soil health and crop safety [79].

Previously, Singh et al. [80] assessed the ecological risks of heavy metals in riverine sediments and found moderate contamination, primarily due to Ni, Pb, and Zn, indicating potential threats to ecosystem health. Tong et al. [55] conducted a health risk assessment of heavy metals in highland barley and wheat, reporting THQ and HI values below 1, suggesting no immediate risk to adult consumers. Chowdhury et al. [81] also evaluated health risks associated with Pb, Cr, Ni, and Fe, using EDI, THQ, HI, and cancer risk metrics. While most EDI values were below MTDI, the total THQ exceeded 1, indicating potential non-carcinogenic effects. The total HI was considerably high, and TCR values for Pb, Cd, and Cr surpassed the carcinogenic threshold, identifying fruit, root, and stem vegetables as major contributors.

## 4. Conclusions

This study showed that irrigation with partially treated paper mill effluent significantly increases the concentration of heavy metals, particularly Cd and Cr, in agricultural soils and wheat crops. Although the calculated health risk indices remain below the threshold of concern, their elevated concentrations and cumulative effects, especially in the context of long-term exposure, pose a potential health risk—mostly for children. These findings showed the need for efficient regulation and pre-treatment of industrial effluents before agricultural application. Moreover, policy frameworks must be enforced to monitor and manage effluent discharge and promote sustainable agricultural practices that safeguard both crop quality and consumer health. This study is limited to a single crop (wheat), which may not fully represent metal uptake dynamics across diverse agricultural systems. Additionally, potential interactions among co-occurring heavy metals were not explicitly modeled, which could influence accumulation patterns and toxicity. Future research should address polymetallic interactions and include multiple crop species to improve risk characterization. Also, future research should focus on remediation strategies, such as phytoremediation or soil amendments, and explore crop-specific tolerance to heavy metal stress.

## Figures and Tables

**Table 1:** Characteristics of irrigation water sources (borewell and paper mill effluent) of the study area.

Parameters	Irrigation Sources		Student’s *t*-Test		Limit for Surface Disposal *	Limit for Inland Irrigation **
	Control (Borewell)	Paper Mill Effluent	*t*-Value	*p*-Value		
pH	7.52 ± 0.03	8.62 ± 0.06	15.37	<0.05	5.50–9.00	5.50–9.00
EC (dS/m)	0.97 ± 0.02	6.55 ± 0.03	76.97	<0.05	NA	NA
TDS (mg/L)	103.03 ± 2.01	1115.29 ± 3.34	302.5	<0.05	1900.00	NA
BOD (mg/L)	3.17 ± 0.02	634.84 ± 3.97	744.69	<0.05	100.00	30.00
COD (mg/L)	7.14 ± 0.01	1254.45 ± 4.73	857.68	<0.05	250.00	250.00
TKN (mg/L)	6.19 ± 0.02	93.92 ± 3.19	122.73	<0.05	100.00	100.00
P (mg/L)	3.54 ± 0.03	64.56 ± 3.92	70.93	<0.05	NA	NA
Na (mg/L)	16.18 ± 1.13	122.63 ± 2.75	37.19	<0.05	NA	NA
K (mg/L)	8.18 ± 1.15	80.06 ± 1.68	33.88	<0.05	NA	NA
Cd (mg/L)	0.09 ± 0.02	0.42 ± 0.03	9.24	<0.05	2.00	2.00
Cr (mg/L)	0.15 ± 0.02	2.04 ± 0.06	28.47	<0.05	2.00	NA
Cu (mg/L)	0.02 ± 0.01	1.58 ± 0.17	42.36	<0.05	3.00	3.00
Fe (mg/L)	1.03 ± 0.02	3.26 ± 0.16	62.3	<0.05	1.00	3.00
Mn (mg/L)	0.83 ± 0.02	2.16 ± 0.02	104.45	<0.05	1.00	2.00
Zn (mg/L)	0.53 ± 0.03	3.65 ± 0.33	28.59	<0.05	15.00	5.00

Values are mean ± standard deviation of 20 samples; *p*-values < 0.05 indicate highly significant differences using a two-tailed *t*-test; NA: not available; *: Central Pollution Control Board of India (CPCB); **: Bureau of Indian Standards (BIS).

**Table 2:** Characteristics of soils irrigated with borewell and paper mill effluent in the study area.

Parameters	Irrigated Soils		Student’s *t*-Test		Agricultural Soil Limits *
	Control (Borewell)	Paper Mill Effluent	*t*-Value	*p*-Value	
pH	7.32 ± 0.13	8.77 ± 0.05	10	<0.05	6.0–8.5
EC (dS/m)	1.93 ± 0.05	4.60 ± 0.21	12.01	<0.05	<4.0
OM (%)	1.59 ± 0.05	3.80 ± 0.06	28.28	<0.05	NA
TKN (mg/kg)	107.48 ± 5.62	210.92 ± 0.93	17.5	<0.05	NA
P (mg/kg)	25.65 ± 3.02	90.21 ± 1.77	18.76	<0.05	NA
Na (mg/kg)	92.28 ± 2.07	191.99 ± 4.37	21.62	<0.05	<230
K (mg/kg)	63.99 ± 2.97	142.40 ± 2.08	35.01	<0.05	NA
Cd (mg/kg)	0.21 ± 0.02	3.08 ± 0.03	119.6	<0.05	1–3
Cr (mg/kg)	0.33 ± 0.02	5.58 ± 0.03	303.26	<0.05	50 (total)
Cu (mg/kg)	0.05 ± 0.01	7.05 ± 0.04	247.49	<0.05	135
Fe (mg/kg)	11.09 ± 0.02	41.94 ± 0.05	328.83	<0.05	Not Established
Mn (mg/kg)	1.72 ± 0.03	7.78 ± 0.06	254.56	<0.05	1500–2000
Zn (mg/kg)	1.19 ± 0.03	9.13 ± 0.03	262.63	<0.05	300

Values are mean ± standard deviation of 20 samples; *p*-values < 0.05 indicate highly significant differences using a two-tailed *t*-test; *: safe limits recommended by USEPA, FAO, and CPCB; NA: not available.

**Table 3:** Impact of irrigated with borewell and paper mill effluent on growth attribute of wheat.

Parameters	Irrigation Source		Student’s *t*-Test	
	Control (Borewell)	Paper Mill Effluent	*t*-Value	*p*-Value
Total chlorophyll content (mg/g fwt.)	4.86 ± 0.05	5.57 ± 0.04	49.23	<0.05
Plant height (cm)	86.28 ± 4.17	94.22 ± 6.94	4.43	<0.05
Leaf length (cm)	17.52 ± 0.59	22.08 ± 0.78	20.91	<0.05
Leaf width (cm)	0.32 ± 0.03	0.38 ± 0.05	4.61	<0.05
Leaf area (cm^3^)	4.17 ± 0.50	6.37 ± 1.05	8.74	<0.05

Values are mean ± standard deviation of 20 samples; *p*-values < 0.05 indicate highly significant differences using a two-tailed *t*-test; fwt.: fresh weight.

**Table 4:** Heavy metal accumulation (mg/kg) in root, stem, leaves, and grains in wheat during irrigated with borewell and paper mill effluent in the study area.

Irrigation Source	Heavy Metals	Wheat Tissues			
		Root	Stem	Leaves	Grain
Control (borewell)	Cd (mg/kg)	0.09 ± 0.01 aA	0.08 ± 0.02 aA	0.05 ± 0.02 bA	0.04 ± 0.01 bA
	Cr (mg/kg)	0.28 ± 0.01 aA	0.25 ± 0.02 aA	0.15 ± 0.03 bA	0.12 ± 0.01 cA
	Cu (mg/kg)	1.72 ± 0.02 aA	0.45 ± 0.04 bA	0.25 ± 0.02 cA	0.06 ± 0.02 dA
	Fe (mg/kg)	15.72 ± 0.12 aA	11.61 ± 0.10 bA	8.96 ± 0.04 cA	6.63 ± 0.09 dA
	Mn (mg/kg)	12.33 ± 0.10 aA	9.79 ± 9.79 aA	5.85 ± 0.03 bA	3.61 ± 0.17 cA
	Zn (mg/kg)	15.64 ± 0.04 aA	11.58 ± 0.05 bA	6.55 ± 0.02 cA	2.43 ± 0.02 dA
Paper mill effluent	Cd (mg/kg)	0.21 ± 0.02 aB	0.13 ± 0.01 bB	0.10 ± 0.01 cB	0.05 ± 0.01 dB
	Cr (mg/kg)	0.19 ± 0.02 aB	0.15 ± 0.03 aB	0.12 ± 0.02 aB	0.07 ± 0.01 bB
	Cu (mg/kg)	8.52 ± 0.02 aB	5.39 ± 0.03 bB	3.24 ± 0.02 cB	1.09 ± 0.02 dB
	Fe (mg/kg)	45.76 ± 0.29 aB	35.41 ± 0.02 bB	23.71 ± 0.34 cB	9.74 ± 0.14 dB
	Mn (mg/kg)	35.34 ± 0.07 aB	24.80 ± 0.14 bB	13.25 ± 0.08 cB	7.13 ± 0.02 dB
	Zn (mg/kg)	23.24 ± 0.07 aB	18.25 ± 0.07 bB	9.05 ± 0.04 cB	4.17 ± 0.02 dB

Values are mean ± standard deviation of 20 samples; Different small letters (a, b, c, d) denote significant differences among tissues within a treatment and metal (row-wise); capital letters (A, B) denote significant differences between irrigation sources for the same tissue and metal (column-wise) at *p* < 0.05, based on Tukey’s HSD test.

**Table 5:** Bioaccumulation factor (Bf) of heavy metals in wheat irrigated with borewell and paper mill effluent.

Heavy Metals	Irrigation Source	Bf Root	Bf Stem	Bf Leaves	Bf Grain
Cd	Control (borewell)	0.41 ± 0.02 a	0.37 ± 0.01 a	0.22 ± 0.01 a	0.17 ± 0.01 a
	Paper mill effluent	0.07 ± 0.01 b	0.04 ± 0.01 b	0.03 ± 0.01 b	0.02 ± 0.00 b
Cr	Control (borewell)	0.87 ± 0.03 a	0.76 ± 0.02 a	0.47 ± 0.01 a	0.36 ± 0.01 a
	Paper mill effluent	0.03 ± 0.01 b	0.03 ± 0.00 b	0.02 ± 0.00 b	0.01 ± 0.00 b
Cu	Control (borewell)	32.31 ± 1.02 a	8.38 ± 0.25 a	4.63 ± 0.15 a	1.19 ± 0.06 a
	Paper mill effluent	1.21 ± 0.05 b	0.77 ± 0.03 b	0.46 ± 0.02 b	0.16 ± 0.01 b
Fe	Control (borewell)	1.42 ± 0.05 a	1.05 ± 0.04 a	0.81 ± 0.03 a	0.60 ± 0.02 a
	Paper mill effluent	1.09 ± 0.04 b	0.84 ± 0.03 b	0.57 ± 0.02 b	0.23 ± 0.01 b
Mn	Control (borewell)	7.17 ± 0.21 a	5.69 ± 0.18 a	3.40 ± 0.12 a	2.10 ± 0.08 a
	Paper mill effluent	4.54 ± 0.15 b	3.19 ± 0.10 b	1.70 ± 0.06 b	0.92 ± 0.03 b
Zn	Control (borewell)	13.18 ± 0.45 a	9.76 ± 0.31 a	5.52 ± 0.18 a	2.05 ± 0.07 a
	Paper mill effluent	2.54 ± 0.08 b	2.00 ± 0.06 b	0.99 ± 0.03 b	0.46 ± 0.02 b

Values are mean ± standard deviation of 20 samples; significance letters (a, b) indicate statistical differences between control (borewell) and paper mill effluent irrigation treatments within each tissue for each metal (at *p* < 0.05 using Tukey’s post hoc test).

**Table 6:** Translocation factor (Tf) of heavy metals in wheat irrigated with borewell and paper mill effluent.

Heavy Metals	Irrigation Source	Tf Stem	Tf Leaves	Tf Grain
Cd	Control (borewell)	0.88 ± 0.04 a	0.54 ± 0.02 a	0.42 ± 0.02 a
	Paper mill effluent	0.62 ± 0.03 b	0.46 ± 0.02 b	0.25 ± 0.01 b
Cr	Control (borewell)	0.87 ± 0.03 a	0.54 ± 0.02 b	0.41 ± 0.01 a
	Paper mill effluent	0.78 ± 0.03 b	0.64 ± 0.02 a	0.38 ± 0.01 b
Cu	Control (borewell)	0.26 ± 0.01 b	0.14 ± 0.01 b	0.04 ± 0.00 b
	Paper mill effluent	0.63 ± 0.03 a	0.38 ± 0.02 a	0.13 ± 0.01 a
Fe	Control (borewell)	0.74 ± 0.03 a	0.57 ± 0.02 a	0.42 ± 0.02 a
	Paper mill effluent	0.77 ± 0.03 a	0.52 ± 0.02 b	0.21 ± 0.01 b
Mn	Control (borewell)	0.79 ± 0.03 a	0.47 ± 0.02 a	0.29 ± 0.01 a
	Paper mill effluent	0.70 ± 0.03 b	0.37 ± 0.01 b	0.20 ± 0.01 b
Zn	Control (borewell)	0.74 ± 0.03 b	0.42 ± 0.02 a	0.16 ± 0.01 b
	Paper mill effluent	0.79 ± 0.03 a	0.39 ± 0.02 b	0.18 ± 0.01 a

Values are mean ± standard deviation of 20 samples; superscript significance letters (a, b) indicate statistical differences between control (borewell) and paper mill effluent irrigation treatments within each tissue for each metal (at *p* < 0.05 using Tukey’s post hoc test).

**Table 7:** DIM, HRI and THQ-based health risk assessment of heavy metals accumulation in wheat grains irrigated with borewell water and paper mill effluent.

Irrigation Source	Exposure Group	Index	Heavy Metal					
			Cd	Cr	Cu	Fe	Mn	Zn
Borewell water	Adult	DIM	0.00020	0.00050	0.00030	0.02840	0.01550	0.01040
		HRI	0.15700	0.10000	0.00700	0.04100	0.11100	0.03500
		THQ	0.00016	0.00010	0.00001	0.00004	0.00011	0.00004
		∑THQ	0.00046					
	Children	DIM	0.00020	0.00070	0.00040	0.03820	0.02080	0.01400
		HRI	0.21200	0.13500	0.00900	0.05500	0.14900	0.04700
		THQ	0.00021	0.00013	0.00001	0.00005	0.00015	0.00005
		∑THQ	0.00060					
Paper mill effluent	Adult	DIM	0.00020	0.00030	0.00470	0.04170	0.03050	0.01790
		HRI	0.22900	0.06300	0.11700	0.06000	0.21800	0.06000
		THQ	0.00023	0.00006	0.00012	0.00006	0.00022	0.00006
		∑THQ	0.00075					
	Children	DIM	0.00030	0.00040	0.00630	0.05620	0.04110	0.02410
		HRI	0.30800	0.08500	0.15800	0.08000	0.29400	0.08000
		THQ	0.00031	0.00008	0.00016	0.00008	0.00029	0.00008
		∑THQ	0.00100					

Values are mean ± standard deviation of 20 samples.

## Data Availability

The original contributions presented in this study are included in the article. Further inquiries can be directed to the corresponding author.
